# The Patients' Column.—III. A London and a Provincial General Hospital Experience

**Published:** 1914-05-02

**Authors:** 


### THE PATIENTS' COLUMN.*

### III. A London and a Provincial General Hospital Experience.

I have been this year, for the first time in my life, a patient in two large and well-known institutions. The first was a large general hospital in London, and I arrived there one day at noon. I was admitted straight to the ward, and was received by the sister and given a chair by the fire. It seemed to be a very busy time in the ward just then. Dinners had just been served, and probationers and staff-nurse alike were busily feeding the helpless and seeing that all. were satisfactorily served. I had had lunch before coming in, but they did not forget to ask me if I would like some food or a glass of milk. After the patients had finished the meal and all was cleared away basins were given out, and again I saw the nurses washing the hands and faces of the helpless ones, whilst those who were able to perform the same office for themselves had everything put in readiness to their hands. Then some of the staff disappeared to their own dinner, and the remainder continued the usual ward work, sweeping the floor, straightening the beds, etc. It seemed that now there was leisure to attend to me, and a pleasant-faced nurse came to take my temperature, after which I was taken to the bathroom.

"The Rule on Admission."

It seems to me a pity that nurses may not exercise their own judgment as to how far these operations are necessary with "admissions," for, in spite of the fact that I, knowing the day before that I was to come to this institution, had been able both to take a bath and wash my hair before leaving home I had to submit to having all the usual operations, because "it was the rule." However, I proceeded to bed, and there remained for nearly eleven weeks! One thing which troubled me very much was the harassed state of the nurses. They seemed to have far too much to do, and though their hours were long they could hardly get the work done "to time"; consequently there was often such a bustle and hurry that doors were banged and screens rattled in a way that was very trying to a nervous invalid. The nurses were all, I think, kind-hearted, but many a time they were too busy to perform the little offices which mean so much to helpless patients.

The Balcony and the Night Nurses.

When I had been a day or so there, and finding that I suffered dreadfully from inability to sleep at night, Sister asked me if I would like to be put out on the balcony. After this, a much happier life was mine. I was well wrapped up, and extra blankets tucked over me, and each day, unless the weather was quite impossible, my bed was wheeled out on to the balcony and I stayed there most of the day. Every effort was made to diagnose my disease, and various specialists came to see me; also, whilst in bed, I experienced the attentions of the dentist, and twice had teeth extracted, each time under an anæsthetic. The food was plentiful and good, but was not always so well cooked as one could wish. The night nurses were most kind and sympathetic, and did much to make life more bearable under strange conditions. When I was able to leave the hospital I felt that I was really leaving friends, and I should not again feel the dread that I suppose most patients feel on going into such an institution.

*Previous articles appeared on April 18 and 25.

My second experience was of a somewhat different nature. This time I was admitted to a surgical ward in a smaller institution in a Northern city. My surroundings were far more up to date, but I missed the kindly manner of sister and nurses. They were so busy, and so many more patients passed through their hands.

A Noisy Provincial Ward.

I was too ill this time for the bath and hair-washing, and was got to bed as quickly as possible. My chief worry was the clashing of doors, which seemed to go on incessantly day and night. I have a very dim recollection of the first few days and nights, of surgeons and dressers, sisters and nurses, coming and going; but then, my symptoms being relieved, I began to take more interest in my surroundings, and, allowing for the difference between the habits and customs of Northern and Southern folk, I soon found myself more at home, and here, again, I found much kindness, but little time to exercise it. I stayed a month, and was then discharged on promising to report myself to the surgeon occasionally.

The Scarcity of Beds.

This, in fact, I did, and at all times I found much courtesy from the staff. I only wished that it had been possible to double the number of beds, with adequate nursing staff, that they might find more leisure to get through the day's work, and also that the patients themselves might have been able to stay longer before making room for the next patient, instead, as was often the case, of having to be taken out, by stretcher and ambulance, to finish the period of convalescence at home.

[Note.—Our readers will not fail to note that here again, as in many of the Patients' articles, it is not the food which is criticised, but its cooking and service. An article on "The Service of Patients' Food," with a subtitle, "The Heel of Achilles in Hospital Work," was published in The Hospital of November 22, 1914, page 199.—Ed. The Hospital.]

